# Educational Level and Length of Work Experience as Correlates of Adverse-Event Reporting and Patient-Safety Perception Among Nurses in Croatian General and County Hospitals: A National Cross-Sectional Study

**DOI:** 10.3390/nursrep16070220

**Published:** 2026-06-26

**Authors:** Ivana Herak, Marijana Neuberg, Valentina Vincek, Valentina Novak, Anita Lukić

**Affiliations:** 1 Department of Nursing, University North, University Centre Varaždin, 104. Brigade 3, 42000 Varaždin, Croatia; marijana.neuberg@unin.hr (M.N.); vavincek@unin.hr (V.V.); vanovak@unin.hr (V.N.); lukic.anita@yahoo.com (A.L.); 2 Faculty of Health Sciences, University of Novo Mesto, 8000 Novo Mesto, Slovenia; 3 Varaždin General Hospital, I. Meštrovića bb, 42000 Varaždin, Croatia; 4 Department of Nursing, Bjelovar University of Applied Sciences, Trg Eugena Kvaternika 4, 43000 Bjelovar, Croatia

**Keywords:** adverse events, patient safety, nursing education, work experience, safety perception, safety culture, incident reporting, cross-sectional studies, nursing workforce, Croatia

## Abstract

**Background/Objectives:** Formal level of education and length of professional experience are widely thought to shape both how often nurses report adverse events and how safe they perceive their workplace to be for patients. Large multicentre data from Central and Eastern European secondary-care systems are scarce. This study examined whether educational level and length of work experience are associated with (a) the self-reported frequency of adverse-event reporting and (b) the perceived level of patient safety in a national sample of nurses working in Croatian general and county hospitals. **Methods:** We conducted a cross-sectional, multicentre survey in 2023 across all 22 general and county hospitals in the Republic of Croatia. A 99-item paper questionnaire—81 items distributed across six previously validated scales (Cronbach’s α 0.730–0.951)—was distributed proportionally to the eligible nursing workforce (*N* = 6661). Of the 1657 questionnaires distributed, 1518 were returned fully completed (response rate 91.6%). Two outcomes were examined in parallel: self-reported frequency of adverse-event reporting in the past 12 months and global perceived level of patient safety on the respondent’s ward. Group comparisons used Pearson’s chi-square and Kruskal–Wallis H tests; effect sizes were estimated using the phi coefficient and Cramér’s V. A multivariable logistic regression was additionally fitted to test whether the associations held after mutual adjustment. The study followed the STROBE reporting guidelines. **Results:** Educational level was associated with the frequency of adverse-event reporting (χ^2^ = 32.54, df = 8, *p* < 0.001; Cramér’s V = 0.10) and with safety perception (χ^2^ = 16.07, df = 8, *p* = 0.041; V = 0.07). Length of total work experience was associated with reporting (χ^2^ = 23.01, df = 12, *p* = 0.028; V = 0.07) and safety perception (χ^2^ = 34.84, df = 12, *p* < 0.001; V = 0.09). After mutual adjustment, bachelor’s (adjusted OR 1.41, 95% CI 1.10–1.81; *p* = 0.006) and master’s or doctoral education (adjusted OR 1.82, 95% CI 1.27–2.62; *p* = 0.001) remained associated with active reporting relative to secondary-level qualification. A notable finding was that 930 nurses (61.3%) had not filed any adverse-event report in the past 12 months. **Conclusions:** Educational level and length of work experience are associated with both the reporting of adverse events and the perception of patient safety among Croatian hospital nurses, but the two characteristics relate to the outcomes in different ways. Higher education is associated with more frequent reporting and a more favourable view of patient safety. Length of experience shows a mid-career peak in reporting but a less favourable perception of safety in late-career nurses. Continued investment in formal nursing education, together with mid- and late-career retention strategies, may strengthen both reporting behaviour and the lived safety culture in Central and Eastern European secondary-care systems. The findings inform nursing leadership, continued education planning, and national patient-safety policy in Central and Eastern European secondary-care systems.

## 1. Introduction

Adverse-event reporting and the perception of patient safety are two closely related but conceptually distinct dimensions of hospital safety culture. The first is a behaviour—the documented willingness of clinicians to formally record events that did, or could have, harmed a patient. The second is an attitude—the global judgement a clinician forms about how safe their workplace is for the patients in their care. The World Health Organization’s Global Patient Safety Action Plan 2021–2030 treats both as priority targets for improvement and explicitly calls for measurement systems in which reporting and perception are tracked together rather than in isolation [1]. Nurses, who provide the largest share of bedside care and witness most medication, procedural, and care-coordination errors, occupy a central role in this measurement architecture [2,3].

Despite this central role, under-reporting by hospital nurses remains persistent and well documented [4,5]. Systematic reviews and qualitative syntheses consistently group the underlying barriers into three layers: organisational factors such as a punitive culture, complex reporting pathways and absent feedback [4,6]; team-level factors such as poor communication and limited management visibility [3,7]; and individual-level factors, of which fear of blame, time pressure, and uncertainty about what counts as a reportable event are the most frequently cited [4,5]. Within this third layer, two sociodemographic characteristics are repeatedly proposed but rarely tested at scale: formal level of education and length of professional experience [8,9].

The link between education and safety culture has been explored mostly through the lens of nursing leadership and the practice environment. Studies in *Nursing Reports* and adjacent nursing journals have shown that workforces with a higher proportion of bachelor’s- and master’s-prepared nurses tend to operate in environments where reporting is more frequent and where the wider safety climate is rated more favourably by staff [10,11,12]. Continued education and curriculum studies have, in parallel, shown that targeted training in patient-safety competencies is associated with stronger self-efficacy in error recognition and a higher stated intention to report [13,14]. By contrast, the role of length of professional experience has been less consistently described. Some single-hospital studies report a positive linear association between years of practice and reporting; others find a non-linear, mid-career peak; and qualitative work suggests that the most experienced nurses may, in fact, disengage from formal reporting because of habituation, perceived futility, or pre-retirement attitudes [4,5,9]. Conceptually, these patterns are consistent with Reason’s model of human error and organisational safety, which frames individual reporting behaviour as the joint product of personal competence (developed through education and clinical experience) and the surrounding system’s tolerance for honest disclosure [4]. To our knowledge, no national multicentre study has examined how education and experience relate to reporting and safety perception in parallel within a single Central or Eastern European workforce.

Most of the available evidence has, in addition, been generated in high-income North American and Western European systems. Central and Eastern European secondary-care hospitals, where the nursing workforce is more heterogeneous in formal education and where reporting infrastructure is still maturing, are under-represented in this literature. Croatia is a useful setting in which to address this gap. Croatian nurses span three formal qualification levels (secondary medical school, SSS; Bachelor of Nursing, VŠS; and master’s or doctoral level, VSS+DR), and adverse-event reporting is legally mandated for sentinel events but operationally heterogeneous across institutions [15]. Existing Croatian work has been confined to psychometric validation of the Hospital Survey on Patient Safety Culture and to single- or three-hospital descriptive studies [15]; multicentre evidence on the demographic correlates of reporting behaviour and safety perception has not previously been published in a peer-reviewed nursing journal.

The present study, drawn from a national doctoral survey of all 22 general and county hospitals in Croatia, was designed to address this gap. Its specific aims were (i) to describe the self-reported frequency of adverse-event reporting and the perceived level of patient safety in this national workforce; and (ii) to test, in parallel, whether educational level and length of work experience are associated with each of these two outcomes. Following the doctoral protocol from which this analysis is derived, four pre-specified hypotheses were examined. For consistency with the larger doctoral analysis, of which the present manuscript reports a focused subset, the original numbering of the hypotheses (H2a–H3b) has been preserved; H1 in the doctoral protocol concerned organisational and unit-level predictors that fall outside the scope of the present paper:

**H2a.**  *Self-reported frequency of adverse-event reporting differs significantly between groups of nurses defined by formal educational level.*

**H2b.**  *Self-reported frequency of adverse-event reporting differs significantly between groups of nurses defined by length of total work experience.*

**H3a.**  *Perceived level of patient safety differs significantly between groups of nurses defined by formal educational level.*

**H3b.**  *Perceived level of patient safety differs significantly between groups of nurses defined by length of total work experience.*

## 2. Materials and Methods

### 2.1. Study Design and Reporting Guideline

A national, cross-sectional, multicentre, quantitative survey was conducted in 2023 in all 22 general and county hospitals in the Republic of Croatia. The study followed the STROBE Statement for cross-sectional studies (Supplementary Material S1). The aim, population, hypotheses, and statistical plan were specified prior to data collection as part of the doctoral research protocol approved by the participating institutions.

### 2.2. Setting and Population

The eligible population comprised all registered nurses and nursing technicians employed on inpatient wards of the 22 general and county hospitals in Croatia (*N* = 6661). General and county hospitals form the secondary level of the Croatian public-healthcare system and cover surgical, internal medicine, gynaecology/obstetrics, paediatric, psychiatric, intensive-care and anaesthesiology departments. Tertiary university hospitals, special hospitals, and primary-care institutions were excluded.

### 2.3. Sample Size and Sampling

A target sample of 25% of the eligible population (1657 nurses) was allocated proportionally to each hospital’s nursing workforce. Within each hospital, the chief nursing officer distributed the paper questionnaire to nurses on inpatient wards using a convenience approach during regular shifts. Of the 1657 questionnaires distributed, 1518 were returned fully completed (response rate 91.6%); this sample is well above the minimum required to detect a small-to-medium effect (φ = 0.10) at α = 0.05 with 80% power for chi-square tests on the relevant 3 × 3 and 4 × 3 contingency tables.

### 2.4. Instrument

The 99-item paper questionnaire combined a sociodemographic and professional section (18 items) with six validated scales (81 items): (1) interpersonal relationships (16 items; 5 reverse-coded); (2) management attitude towards patient safety (4 items; 2 reverse-coded); (3) communication in the work environment (6 items; 1 reverse-coded); (4) management approach to patient safety (10 items; 6 reverse-coded); (5) personal safety experience (11 items; 2 reverse-coded); and (6) frequency of inappropriate nursing care practices (34 items, no reverse-coding). The principal outcome for the present analysis—self-reported frequency of adverse-event reporting in the past 12 months—was captured on a five-point ordered scale (none; 1–2; 3–5; 6–10; >10 reports). Internal consistency in this sample was satisfactory to high across all six scales (Cronbach’s α 0.730–0.951). The English-language version is provided as Supplementary Material S3.

### 2.5. Variables

Two outcomes were examined in parallel. The first was self-reported frequency of adverse-event reporting in the past 12 months, captured on a five-point ordered scale (none; 1–2; 3–5; 6–10; ≥11 reports). The second was the respondent’s global perception of patient safety on their ward, captured on a five-point ordinal scale (none; poor; acceptable; very good; and excellent). The two main independent variables were formal educational level (secondary medical school [SSS]; Bachelor of Nursing [VŠS]; master’s or doctoral degree [VSS+DR]) and length of total work experience in healthcare (≤10; 11–20; 21–30; ≥31 years). Length of service in the current hospital, sex, age band, hospital department, the workplace communication scale score and the management approach scale score were retained as descriptive variables.

### 2.6. Statistical Analysis

Descriptive statistics summarised demographic and professional characteristics. Group comparisons used Pearson’s chi-square tests for categorical contingency tables (with validity verified by ensuring that fewer than 20% of expected frequencies were below 5; one test failed this criterion and is reported with the corresponding caveat). For ordinal and non-normally distributed continuous outcomes, the Mann–Whitney U test (two-group comparisons) and the Kruskal–Wallis H test (more than two groups) were used. Effect sizes were quantified with the phi coefficient and Cramér’s V, interpreted as follows: 0.00–0.15 very weak; 0.15–0.20 weak; 0.20–0.25 moderate; 0.25–0.30 moderately strong; 0.30–0.35 strong; and 0.35–0.40 very strong. The threshold for statistical significance was *p* < 0.05; results are reported as *p* < 0.05 (*), *p* < 0.01 (**), and *p* < 0.001 (***). Analyses were performed in IBM SPSS Statistics version 27. To test whether the bivariate associations held after mutual adjustment, a multivariable binary logistic regression model was additionally fitted, with active adverse-event reporting (≥1 report in the past 12 months) as the dependent variable. Independent variables entered simultaneously were educational level (SSS as reference; VŠS; VSS+DR), length of total work experience (≤ 10 years as reference; 11–20; 21–30; ≥ 31), age category, sex, and the mean score on the ‘frequency of safety reporting’ scale. Model fit was assessed using the Hosmer–Lemeshow goodness-of-fit test, and multicollinearity was assessed using the variance inflation factor (VIF), with VIF > 5 considered indicative of concern. For the pre-specified sensitivity analyses, Bonferroni correction was applied to the four primary chi-square tests (adjusted α = 0.0125), and Mantel–Haenszel linear-by-linear trend tests were used to confirm the monotonic gradients observed in cross-tabulations.

### 2.7. Ethical Considerations

The study was conducted in accordance with the Declaration of Helsinki and was approved by the Institutional Review Boards/Ethics Committees of all 22 participating hospitals. Representative approval codes include General Hospital Varazdin 02/191/111-2022; County Hospital Cakovec 01-4763/1/2022; County Hospital Nasice 01-87/2-2023; the full list of approval codes is provided as Supplementary Material S2. All approvals were obtained prior to data collection. Participation was voluntary and anonymous; written informed consent was obtained from all participants prior to questionnaire completion. The study population consisted of registered nurses and nursing technicians; no patients, service users, carers, or members of the public were involved in the design, conduct, or reporting of this research.

## 3. Results

### 3.1. Sample Characteristics

Of the 1657 distributed questionnaires, 1518 were returned fully completed (response rate 91.6%). Most participants were women (*n* = 1222; 80.5%), 209 were men (13.8%), and 87 (5.7%) preferred not to disclose their sex. More than half of the samples (*n* = 891; 58.7%) were younger than 40 years. By educational level, 883 participants (58.2%) had completed secondary medical school (SSS), 476 (31.4%) held a bachelor’s degree in nursing (VŠS), 147 (9.7%) held a master’s degree, and approximately 1% held a doctoral degree (master’s and doctoral participants were pooled into a single VSS+DR category for the present analyses). The largest groups worked in surgery and internal medicine departments (combined: 64% of the sample). Most participants (41%) had less than 10 years of total work experience in the healthcare system; the mean total experience was 16 years.

### 3.2. Instrument Reliability

Internal consistency was confirmed for all six scales in the main study (Table 1). Cronbach’s α ranged from 0.730 (management attitude towards patient safety) to 0.951 (frequency of inappropriate nursing care practices), corresponding to satisfactory-to-high reliability across all conceptual domains of the instrument.

### 3.3. Educational Level and Adverse-Event Reporting (H2a)

Educational level was associated with the self-reported frequency of adverse-event reporting in both the contingency-table framework and the rank-based framework. The Pearson chi-square test for the three-by-five table of educational level was conducted by reporting the frequency returned χ^2^ = 32.54 (df = 8), *p* < 0.001, with a very weak effect size (Cramér’s V = 0.10). The Kruskal–Wallis H test confirmed a monotonic gradient across the three educational groups (Table 2): mean ranks rose from 719.40 in nurses with secondary education (SSS) to 772.93 in bachelor’s-prepared nurses (VŠS) to 836.56 in nurses with a master’s or doctoral qualification (VSS+DR), with H = 15.901 (df = 2), *p* < 0.001. Hypothesis H2a was therefore supported.

### 3.4. Length of Work Experience and Adverse-Event Reporting (H2b)

Length of total work experience in the healthcare system was also associated with reporting frequency, which had a smaller effect than education and a non-monotonic shape. The four-by-five chi-square test returned χ^2^ = 23.01 (df = 12), *p* = 0.028 (Cramér’s V = 0.07). The Kruskal–Wallis H test resulted in H = 9.249 (df = 3), *p* = 0.026, with mean ranks of 718.99 (≤10 years), 783.41 (11–20 years), 779.41 (21–30 years) and 734.39 (≥31 years) (Table 2). Reporting frequency was, thus, highest in the two mid-career bands and lower at both ends of the experience distribution. Hypothesis H2b was therefore supported.

### 3.5. Educational Level and Safety Perception (H3a)

A parallel analysis examined whether educational level was associated with the global perception of patient safety on the respondent’s ward. The three-by-five chi-square test returned χ^2^ = 16.07 (df = 8), *p* = 0.041 (Cramér’s V = 0.07), and the Kruskal–Wallis H test resulted in H = 10.539 (df = 2), *p* = 0.005. Mean ranks rose monotonically across the three educational levels, from 735.29 (SSS) and 775.89 (VŠS) to 844.86 (VSS+DR) (Table 2). Better-educated nurses, thus, reported a more favourable view of patient safety on their ward. Hypothesis H3a was therefore supported.

### 3.6. Length of Work Experience and Safety Perception (H3b)

Length of total work experience was associated with safety perception in the opposite direction to that observed for reporting frequency. The four-by-five chi-square test was the strongest of the four pre-specified tests in this analysis, χ^2^ = 34.84, df = 12, *p* < 0.001; Cramér’s V = 0.09, and the Kruskal–Wallis H test resulted in H = 8.517 (df = 3), *p* = 0.036. Mean ranks were highest in the youngest group (795.08, ≤10 years) and fell progressively through the mid-career groups (733.81 for 11–20 years; 749.86 for 21–30 years) to the lowest value in the most senior nurses (718.17, ≥31 years) (Table 2). The most experienced nurses thus held the least favourable view of patient safety on their ward. Hypothesis H3b was therefore supported.

### 3.7. Summary of Findings

All four pre-specified hypotheses were supported, but the patterns they described were not identical. For the reporting outcome, educational level produced a monotonic, increasing gradient (H2a), whereas length of work experience produced a non-linear mid-career peak (H2b). For the safety-perception outcome, education produced the same monotonic, increasing gradient (H3a), but length of work experience produced a monotonic decreasing gradient—highest in early-career and lowest in late-career nurses (H3b). The two sociodemographic predictors therefore jointly shape both reporting behaviour and safety perception, but operate through different functional dynamics.

### 3.8. Multivariable Logistic Regression

A multivariable binary logistic regression was fitted on *n* = 1398 cases with complete data for all covariates; 529 of these (37.8%) were classified as active reporters (≥1 report). After mutual adjustment, bachelor-level education (adjusted OR 1.41, 95% CI 1.10–1.81; *p* = 0.006) and master’s or doctoral education (adjusted OR 1.82, 95% CI 1.27–2.62; *p* = 0.001) remained independently associated with active reporting relative to secondary-level qualification. Mid-career experience (21–30 years) was also associated with higher odds of active reporting (adjusted OR 1.58, 95% CI 1.14–2.18; *p* = 0.006). Female sex (adjusted OR 0.65, 95% CI 0.49–0.86; *p* = 0.003) and a higher score on the ‘frequency of safety reporting’ scale (adjusted OR 0.76 per scale-point increase, 95% CI 0.65–0.89; *p* < 0.001) were associated with lower odds of active reporting. Nagelkerke R^2^ for the full model was 0.026.

### 3.9. Sensitivity Analyses

Three pre-specified sensitivity analyses were performed to test the robustness of the bivariate findings. First, Bonferroni correction applied to the four primary chi-square tests (adjusted α = 0.0125) confirmed that H2a, H2b, and H3b remained significant; H3a (*p* = 0.041) became borderline under the stricter threshold. Second, Mantel–Haenszel linear-by-linear trend tests confirmed the monotonic gradients across educational levels for both reporting (χ^2^_trend = 21.34, df = 1, *p* < 0.001) and safety perception (χ^2^_trend = 11.82, df = 1, *p* < 0.001). Third, the Hosmer–Lemeshow goodness-of-fit test for the logistic regression model indicated adequate fit (χ^2^ = 4.53, df = 8, *p* = 0.806), and the maximum VIF was 1.22, which is well below the conventional threshold of five, indicating no problematic multicollinearity among the independent variables.

## 4. Discussion

In this national, multicentre survey of 1518 nurses working across all 22 general and county hospitals in Croatia, formal level of education and length of total work experience were each independently associated with both the self-reported frequency of adverse-event reporting and the perceived level of patient safety. The two predictors did not, however, act in the same way. Education produced a clean, monotonic, increasing gradient for both outcomes: better-educated nurses reported more events and perceived their wards as safer. Length of experience produced a more complex pattern—a mid-career peak in reporting, paired with a progressive decline in safety perception from the youngest to the most senior nurses. Taken together, these results align with and extend the international literature on the determinants of nurses’ reporting behaviour and safety culture [4,5,6,7,12].

### 4.1. Education as a Cross-Cutting Predictor (H2a, H3a)

The most consistent finding in our data was the role of formal educational level. Across the three Croatian qualification groups (secondary, bachelor’s, master’s, or doctoral), the same monotonic gradient appeared on both outcomes: higher education was associated with more frequent reporting (H2a) and with a more favourable view of patient safety on the respondent’s ward (H3a). This dual gradient is conceptually important. It mirrors evidence from previous studies in *Nursing Reports* and adjacent nursing journals that link a higher proportion of bachelor’s- and master’s-prepared nurses to a stronger safety climate and to more mature reporting practices [10,11], and it is consistent with the concept-analytic literature, which positions formal education as an antecedent of the positive nursing practice environment [12].

In the patient-safety literature, the simultaneous presence of high reporting and high perceived safety is generally interpreted as a protective rather than a complacent pattern, because it reflects active surveillance rather than passive reassurance [4,7,12]. The fact that this pattern was detectable in our national Croatian data on individual self-reports from a large multicentre sample suggests that the educational gradient is sufficiently robust and can register even in a workforce where reporting infrastructure remains operationally heterogeneous. For Croatian nursing leadership, this is an actionable finding: continued investment in bachelor- and master-level nursing qualifications can reasonably be expected to improve both the reporting behaviour and the lived safety culture of the workforce [13,14].

### 4.2. The Mid-Career Peak in Reporting (H2b)

For length of work experience, the relationship with reporting was non-monotonic and best described as a mid-career peak: nurses with 11–20 and 21–30 years of total experience reported events more frequently than either early-career (≤10 years) or late-career (≥31 years) colleagues. The same pattern has been described in qualitative and systematic review work on speak-up behaviours, where early-career nurses are typically deterred by fear of blame and uncertainty about what counts as a reportable event, and late-career nurses are inhibited by habituation, perceived futility of reporting, or gradual pre-retirement disengagement [4,5,9]. The clinical-leadership implication is that strategies to promote reporting cannot reasonably target a single career stage. Early-career nurses appear to need psychological-safety and just-culture support to begin reporting in the first place, while late-career nurses appear to need engagement and renewal of interventions if their reporting is to be sustained [13,14].

### 4.3. The Inverse Experience Gradient in Safety Perception (H3b)

For the second outcome—perceived level of patient safety—length of experience produced a markedly different pattern. Mean ranks fell progressively from the youngest band (≤10 years; 795.08) to the two mid-career bands and the most experienced nurses (≥31 years; 718.17). In other words, the longer a Croatian nurse had been working in the system, the less favourably they tended to perceive the safety of their ward. We interpret this finding cautiously, but it is striking. It is consistent with two well-described mechanisms in the safety-culture literature. A complementary explanation comes from research linking nurse burnout to safety culture: cumulative occupational fatigue erodes safety perceptions over time, an effect that has been shown to be moderated by structural empowerment at the unit level [16]. The first is calibration: experienced nurses, who have witnessed more near-misses and adverse events over the course of their careers, may rate workplace safety against a more demanding internal benchmark than colleagues earlier in their professional life [4,5,9]. The second is moral distress and burnout: longitudinal nursing-workforce research has repeatedly shown that prolonged exposure to understaffing, workload pressure and unresolved adverse events erodes the safety perception of senior staff [9,17].

The juxtaposition of H2b and H3b is, in our view, the single most policy-relevant signal in this analysis. Mid-career nurses are simultaneously the most active reporters (H2b) and are a group whose safety perception has already begun to decline relative to early-career colleagues (H3b). The most senior nurses report less often (H2b) and rate workplace safety least favourably (H3b). Nursing leadership should therefore not interpret a fall in reporting from senior staff as reassurance about safety; on the contrary, in our data, the two trajectories run in opposite directions. Targeted retention, debriefing, and renewal of interventions for nurses with ≥21 years of experience may be an under-recognised lever for sustaining both reporting and realistic safety perception in this group.

### 4.4. Strengths and Limitations

The principal strengths of this study are its national scope—all 22 general and county hospitals in Croatia were included—the high response rate (91.6%), the use of six previously validated scales with confirmed internal consistency (Cronbach’s α 0.730–0.951), and the pre-specified analytic plan documented in the underlying doctoral protocol. The two outcomes were examined in parallel rather than separately, allowing for a comparative interpretation of education and experience gradients, which constitutes the main contribution of this paper. Limitations include the cross-sectional design, which precludes causal inference—all associations reported here are correlational and cannot establish directionality between sociodemographic characteristics and reporting or safety-perception outcomes; reliance on self-reported frequency rather than verified hospital reporting-system data, which is subject to recall and social-desirability biases; convenience sampling within each hospital; the single-country sampling frame, which limits generalisability to other Central and Eastern European systems with different reporting infrastructures or workforce profiles; and the under-representation of male nurses, which mirrors the Croatian workforce structure but limits sex-stratified analyses. The pooling of master’s and doctoral participants into a single VSS+DR category was necessary because of the small number of doctoral-level nurses; as the Croatian workforce continues to develop, future analyses should examine the doctoral subgroup separately. Finally, one chi-square test marginally exceeded the conventional 20% expected-frequency-below-five threshold and is reported with the corresponding caveat.

### 4.5. Implications for Practice and Policy

Three implications follow from the joint pattern of H2a/H2b and H3a/H3b. First, for nursing leadership and continued education planning, the educational gradient (H2a, H3a) supports continued investment in bachelor- and master-level nursing qualifications as a cross-cutting lever that improves both reporting behaviour and lived safety perceptions. Second, for clinical management at the ward level, divergent experience patterns (H2b versus H3b) suggest that reporting frequency cannot be treated as a standalone indicator of safety culture: a fall in reporting among the most experienced nurses, in our data, coexists with the least favourable safety perception in the workforce. Third, for the national patient-safety policy in Central and Eastern European secondary-care systems, the findings reinforce the case set out by the WHO Global Patient Safety Action Plan 2021–2030 [1], which aimed to combine standardised electronic reporting infrastructure with continued expansion of formal nursing education and targeted mid- and late-career retention programmes.

## 5. Conclusions

In a national, multicentre sample of 1518 nurses working across all 22 Croatian general and county hospitals, formal level of education and length of total work experience were each independently associated with both the self-reported frequency of adverse-event reporting and the perceived level of patient safety, but the two predictors operated along different functional dynamics. Education produced a monotonic, increasing gradient for both outcomes (H2a, H3a): better-educated nurses reported more events and perceived their wards to be safer. Length of experience produced a mid-career peak in reporting (H2b) but a monotonic decrease in safety perception in early- to late-career nurses (H3b). The juxtaposition of these patterns cautions against using reporting frequency as a standalone indicator of safety culture in older cohorts of staff. Within the wider WHO Global Patient Safety Action Plan 2021–2030 agenda, our results support a coordinated investment in formal nursing education, mid- and late-career retention, and standardised reporting infrastructure as complementary, rather than alternative, levers for strengthening organisational safety culture in Central and Eastern European secondary-care systems.

## Figures and Tables

**Table 1:** Internal consistency (Cronbach’s α) of the six scales in the main study (*N* = 1518).

Scale	Items	Reverse-Coded	Cronbach’s α	Interpretation
1. Interpersonal relationships	16	5/16	0.751	Satisfactory
2. Management attitude towards patient safety	4	2/4	0.730	Satisfactory
3. Communication in the work environment	6	1/6	0.781	Satisfactory
4. Management approach to patient safety	10	6/10	0.810	Good
5. Personal safety experience	11	2/11	0.741	Satisfactory
6. Frequency of inappropriate nursing care practices	34	0/34	0.951	High
Total	81	16/81	—	—

Reliability interpretation: α < 0.6 unacceptable; α ≥ 0.7 satisfactory; α ≥ 0.8 good; α ≥ 0.9 high.

**Table 2:** Association of educational level and length of work experience with self-reported adverse-event reporting frequency (H2a, H2b) and with perceived patient safety (H3a, H3b). Pearson chi-square and Kruskal–Wallis H tests; *N* = 1518.

Hypothesis/Association	Subgroup	*n*	Mean Rank	Chi-Square (df, Cramér’s V)	Kruskal–Wallis H (df, *p*)
H2a. Educational level × Reporting	Secondary (SSS)	867	719.40	χ^2^ = 32.54 (df = 8); Cramér’s V = 0.10; *p* < 0.001 ***	H = 15.901 (2); *p* < 0.001 ***
	Bachelor (VŠS)	474	772.93		
	Master’s/Doctoral (VSS+DR)	155	836.56		
H2b. Total work experience × Reporting	≤10 years	612	718.99	χ^2^ = 23.01 (df = 12); Cramér’s V = 0.07; *p* = 0.028 *	H = 9.249 (3); *p* = 0.026 *
	11–20 years	351	783.41		
	21–30 years	296	779.41		
	≥31 years	237	734.39		
H3a. Educational level × Safety perception	Secondary (SSS)	883	735.29	χ^2^ = 16.07 (df = 8); Cramér’s V = 0.07; *p* = 0.041 *	H = 10.539 (2); *p* = 0.005 **
	Bachelor (VŠS)	476	775.89		
	Master’s/Doctoral (VSS+DR)	159	844.86		
H3b. Total work experience × Safety perception	≤10 years	620	795.08	χ^2^ = 34.84 (df = 12); Cramér’s V = 0.09; *p* < 0.001 ***	H = 8.517 (3); *p* = 0.036 *
	11–20 years	355	733.81		
	21–30 years	301	749.86		
	≥31 years	242	718.17		

Note. * *p* < 0.05; ** *p* < 0.01; *** *p* < 0.001. Cramér’s V = effect size for chi-square tests. Subgroup *n* values are based on valid responses for each contingency table; small differences across rows reflect missing values for either the independent or the dependent variable. SSS = secondary medical school; VŠS = Bachelor of Nursing; VSS+DR = master’s or doctoral degree.

## Data Availability

The de-identified dataset analysed during the current study is available from the corresponding author upon reasonable request, subject to the data-sharing provisions of the participating institutions’ ethics approvals.

## Supplementary Materials

The following supporting information can be downloaded at https://www.mdpi.com/article/10.3390/nursrep16070220/s1. Supplementary Material S1—STROBE statement—Checklist of items that should be included in reports of cross-sectional studies. Supplementary Material S2—Ethics committee approvals from participating institutions. Supplementary Material S3—Study Questionnaire (English translation).

## Public Involvement Statement

There was no patient or public involvement in the design, conduct, or reporting of this research. The study population consisted of registered nurses and nursing technicians.

## Guidelines and Standards Statement

This manuscript adheres to the EQUATOR network’s STROBE statement guidelines for the reporting of cross-sectional studies. The completed STROBE checklist is provided as Supplementary Material S1.
