# Supplementary material for: Educational Level and Length of Work Experience as Correlates of Adverse-Event Reporting and Patient-Safety Perception Among Nurses in Croatian General and County Hospitals: A National Cross-Sectional Study
Source: Nurs Rep. 2026 Jun 26;16(7):220. doi: 10.3390/nursrep16070220 (PMC13414478; doi:10.3390/nursrep16070220)
Supplement: Supplementary file 1 [file nursrep-16-00220-s001.zip › SupplementaryS1_STROBE+_Checklist.pdf]

Supplementary Material S1

STROBE Statement — Checklist of items that should be included in reports of cross-sectional studies

Manuscript: *"Educational Level, Work Experience and Self-Reported Frequency of Adverse-Event Reporting Among Nurses in Croatian General and County Hospitals: A National Cross-Sectional Study"*

Authors: Ivana Herak, Marijana Neuberg, Valentina Vincek, Valentina Novak, Anita Lukić

Section / Item	STROBE recommendation	Reported on page / section
**Title and abstract — 1(a)**	Indicate the study's design with a commonly used term in the title or the abstract	Title (p. 1) — "…A National Cross-Sectional Study"; Abstract — Methods (p. 1)
**Title and abstract — 1(b)**	Provide in the abstract an informative and balanced summary of what was done and what was found	Structured abstract (p. 1–2)
**Introduction — Background/rationale 2**	Explain the scientific background and rationale for the investigation being reported	1. Introduction (p. 2–3)
**Introduction — Objectives 3**	State specific objectives, including any prespecified hypotheses	1. Introduction (p. 3) — four pre-specified hypotheses (H2a, H2b, H3a, H3b; numbering follows the doctoral protocol for cross-referencing with companion analyses)
**Methods — Study design 4**	Present key elements of study design early in the paper	2.1. Study Design and Setting (p. 3)
**Methods — Setting 5**	Describe the setting, locations, and relevant dates, including periods of recruitment, exposure, follow-up, and data collection	2.1. Study Design and Setting (p. 3); 2.5. Data Collection (p. 5)
**Methods — Participants 6(a)**	Give the eligibility criteria, and the sources and methods of selection of participants	2.2. Population and Sample (p. 4)
**Methods — Variables 7**	Clearly define all outcomes, exposures, predictors, potential confounders, and effect modifiers	2.3. Instrument and Key Variables (p. 4–5); 2.6. Statistical Analysis (p. 5–6)
**Methods — Data sources/measurement 8***	For each variable of interest, give sources of data and details of methods of assessment (measurement). Describe comparability of assessment methods if there is more than one group	2.3. Instrument (p. 4–5); 2.4. Pilot and Reliability (p. 5)
**Methods — Bias 9**	Describe any efforts to address potential sources of bias	2.5. Data Collection (p. 5); 2.7. Ethical Considerations (p. 6); 4.5. Limitations (p. 12)
**Methods — Study size 10**	Explain how the study size was arrived at	2.2. Population and Sample (p. 4) — Cochran/Raosoft; 25% of N = 6,661
**Methods — Quantitative variables 11**	Explain how quantitative variables were handled in the analyses. If applicable, describe which groupings were chosen and why	2.6. Statistical Analysis (p. 5–6); education in 3 levels (SSS / VŠS / VSS+); experience in 4 strata (≤10 / 11–20 / 21–30 / ≥31 years)
**Methods — Statistical methods 12(a)**	Describe all statistical methods, including those used to control for confounding	2.6. Statistical Analysis (p. 5–6)
**Methods — Statistical methods 12(b)**	Describe any methods used to examine subgroups and interactions	2.6. Statistical Analysis (p. 5–6); 3.3. Rank-Based Comparisons (p. 8)
**Methods — Statistical methods 12(c)**	Explain how missing data were addressed	2.6. Statistical Analysis (p. 5–6) — case-wise valid n reported per item

Section / Item	STROBE recommendation	Reported on page / section
**Methods — Statistical methods 12(d)**	If applicable, describe analytical methods taking account of sampling strategy	2.2. Population and Sample (p. 4) — proportional allocation by hospital
**Methods — Statistical methods 12(e)**	Describe any sensitivity analyses	Not applicable — primary descriptive/associational analysis
**Results — Participants 13(a)***	Report numbers of individuals at each stage of study—e.g., numbers potentially eligible, examined for eligibility, confirmed eligible, included in the study, completing follow-up, and analysed	3.1. Participants (p. 6–7)
**Results — Participants 13(b)**	Give reasons for non-participation at each stage	3.1. Participants (p. 6–7) — non-response 8.4%; reasons not individually recorded (anonymous distribution)
**Results — Participants 13(c)**	Consider use of a flow diagram	Not used — single-stage cross-sectional design; counts reported in text
**Results — Descriptive data 14(a)***	Give characteristics of study participants (e.g., demographic, clinical, social) and information on exposures and potential confounders	3.1. Participants (p. 6–7); Table 1 (p. 7)
**Results — Descriptive data 14(b)**	Indicate number of participants with missing data for each variable of interest	Reported as valid n per item across results sections
**Results — Outcome data 15***	Report numbers of outcome events or summary measures	3.2. Education and Reporting (p. 7–8); 3.3. Experience and Reporting (p. 8); Table 2 (p. 8–9)
**Results — Main results 16(a)**	Give unadjusted estimates and, if applicable, confounder-adjusted estimates and their precision	3.2.–3.3. (p. 7–9) — χ^2, φ, Kruskal–Wallis H statistics with df and p
**Results — Main results 16(b)**	Report category boundaries when continuous variables were categorized	2.3. Instrument (p. 4–5); 3.3. (p. 8) — experience strata
**Results — Main results 16(c)**	If relevant, consider translating estimates of relative risk into absolute risk for a meaningful time period	Not applicable — descriptive associational design
**Results — Other analyses 17**	Report other analyses done—e.g., analyses of subgroups and interactions, and sensitivity analyses	3.3. Rank-Based Comparisons (p. 8); Table 2 (p. 8–9)
**Discussion — Key results 18**	Summarise key results with reference to study objectives	4.1. Principal Findings (p. 9–10)
**Discussion — Limitations 19**	Discuss limitations of the study, taking into account sources of potential bias or imprecision	4.5. Limitations (p. 12)
**Discussion — Interpretation 20**	Give a cautious overall interpretation of results considering objectives, limitations, multiplicity of analyses, results from similar studies, and other relevant evidence	4.2.–4.4. Discussion sections (p. 10–12)
**Discussion — Generalisability 21**	Discuss the generalisability (external validity) of the study results	4.4. Practical Implications (p. 11–12); 4.5. Limitations (p. 12)
**Other information — Funding 22**	Give the source of funding and the role of the funders for the present study and, if applicable, for the original study on which the present article is based	Funding statement (back matter)

Note. *An asterisk (*) following an item number indicates that the information should be given separately for cases and controls in case-control studies, or for exposed and unexposed groups in cohort and cross-sectional studies. The STROBE checklist is best used in conjunction with the explanatory article (von Elm E, Altman DG, Egger M, Pocock SJ, Gøtzsche PC, Vandenbroucke JP. The Strengthening the Reporting of Observational Studies in Epidemiology (STROBE) Statement: guidelines for reporting observational studies. PLoS Med. 2007;4(10):e296), available at https://www.strobe-statement.org.*
