# Supplementary material for: Educational Level and Length of Work Experience as Correlates of Adverse-Event Reporting and Patient-Safety Perception Among Nurses in Croatian General and County Hospitals: A National Cross-Sectional Study
Source: Nurs Rep. 2026 Jun 26;16(7):220. doi: 10.3390/nursrep16070220 (PMC13414478; doi:10.3390/nursrep16070220)
Supplement: Supplementary file 1 [file nursrep-16-00220-s001.zip › SupplementaryS2_EthicsApprovals.pdf]

Supplementary File S2

Ethics committee approvals from participating institutions

This study received written approval from the institutional ethics committees of all 22 Croatian general and county hospitals where data collection was performed, as well as from the Ethics Committee for Research Involving Humans of the University of Novo Mesto (Faculty of Health Sciences, Slovenia), under whose doctoral programme the research was conducted. All approvals authorise the cross-sectional survey described in the manuscript entitled "The impact of nursing care procedures on patient safety at the secondary level of healthcare in the Republic of Croatia". Original approval letters are held on file by the corresponding author and are available upon reasonable request to the editorial office.

#	Institution	Approval reference number	Date of approval	Chair / Issuing authority
1	County Hospital Čakovec	01-4763/1/2022	2 December 2022	Dejan Balažin
2	General Hospital of Šibenik-Knin County	01-2318/1-23	1 February 2023	Krešimir Stipaničev
3	General Hospital Vinkovci	01-665/1/23 (Urbroj 665/23)	31 January 2023	Krunoslav Šporčić
4	General Hospital "Hrvatski ponos" Knin	Klasa 510-01/23-01/33; Urbroj 2182-10-17/09-23-2	7 February 2023	Nada Kljajić
5	General Hospital Zabok and Hospital of Croatian Veterans	01-445/2-23	7 February 2023	Bojana Kranjčec
6	National Memorial Hospital "Dr. Juraj Njavro" Vukovar	EP-3/2023; Klasa 510-05/23; Urbroj 107-01/23-1-04	8 February 2023	Mara Jakšić
7	General Hospital Virovitica	Klasa 510-03/23-01/483; Urbroj 2189-43-02/1-23-2	7 February 2023	Jadranko Šegregur
8	General Hospital Pula	Klasa 641-01/23-01/01; Urbroj 2168/01-59-79-112-23-9	9 February 2023	Boris Grdinić
9	General Hospital Dubrovnik	Klasa 021-01/23-05/28; Urbroj 191-1-23-6	14 February 2023	Igor Borić
10	General Hospital Varaždin	02/1-91/111-2022	19 December 2022	Krunoslav Košćak
11	General Hospital "Dr. Josip Benčević" Slavonski Brod	Decision of the Ethics Committee	14 February 2023	Jasminka Kopić
12	General County Hospital Našice	01-87/2-2023	16 February 2023	Marina Hlavati
13	General Hospital Nova Gradiška	Ur.broj 01-900/23	20 February 2023	Darko Kikić
14	General Hospital "Dr. Tomislav Bardek" Koprivnica	Klasa 053-02/23-01/07; Urbroj 2137-84-01-23-2	22 February 2023	Stjepan Gašparić
15	General Hospital and Hospital of Croatian Veterans Ogulin	Broj 01-14/4-1	20 February 2023	President of the Ethics Committee
16	General Hospital Gospić	Ur.broj 2125/53-04-637/23-2	6 March 2023	Mirjana Pećina

#	Institution	Approval reference number	Date of approval	Chair / Issuing authority
17	General Hospital "Dr. Ivo Pedišić" Sisak	Urbroj 2176-125-04-1278-3/23	27 February 2023	Igor Vrga
18	General Hospital Karlovac	18-01.4/1-23	23 February 2023	Zorica Alerić
19	General Hospital Bjelovar	Klasa 053-02/23-02/38; Urbroj 2103-72-12-23-02	3 March 2023	Darko Novalić
20	General County Hospital Pakrac and Hospital of Croatian Veterans	Ur. broj 32-1-198/23-4	29 March 2023	Kristina Milek
21	General County Hospital Požega	Ur.broj 02-7/2-2/1-2-2023	31 March 2023	Goran Šantak
22	General Hospital Zadar	Ur.broj 01-2981/23-3/23	25 April 2023	Klaudia Duka Glavor
–	*University of Novo Mesto, Faculty of Health Sciences, Slovenia (sponsoring institution; Ethics Committee for Research Involving Humans)*	UNM 101/2024	26 November 2024	Nevenka Kregar Velikonja

Notes:

- KLASA / Klasa and URBROJ / Urbroj are mandatory Croatian administrative classification and registry numbers used by public institutions; the format reflects the original document on file.
- All data collection commenced only after the corresponding institutional approval was obtained.
- The University of Novo Mesto (Slovenia) approval covers the doctoral research project as a whole; the 22 Croatian hospitals issued site-specific approvals.
- Where the original approval letter did not display a clearly typed reference number, the document is referred to by the issuing authority and decision date.
