# Supplementary material for: Educational Level and Length of Work Experience as Correlates of Adverse-Event Reporting and Patient-Safety Perception Among Nurses in Croatian General and County Hospitals: A National Cross-Sectional Study
Source: Nurs Rep. 2026 Jun 26;16(7):220. doi: 10.3390/nursrep16070220 (PMC13414478; doi:10.3390/nursrep16070220)
Supplement: Supplementary file 1 [file nursrep-16-00220-s001.zip › SupplementaryS3_Questionnaire_English.pdf]

# Supplementary Material S3

## Study Questionnaire (English translation)

**Manuscript:** *" Educational Level and Length of Work Experience as Predictors of Adverse-Event Reporting and Patient-Safety Perception Among Nurses in Croatian General and County Hospitals: A National Cross-Sectional Study"*

**Authors: Ivana Herak, Marijana Neuberg, Valentina Vincek, Valentina Novak and Anita Lukić**

*Translation note. The original questionnaire was developed and administered in Croatian. Items adapted from the "Patient Safety Culture in the Hospital Survey" (Šklebar, 2014) were used in the original Croatian wording. Items adapted from the "Patient Safety Climate in Healthcare Organizations" questionnaire (Singer, 2006) were translated from English to Croatian and back-translated to English by professional translators (Žerjav School of Foreign Languages); written permission for use and translation was obtained from S. J. Singer. Items 4.1 to 4.6 were originally drafted in Croatian by the first author (Herak, 2022). The English version below was prepared for the present international submission and reflects the meaning of the Croatian items used in the study; the wording of items adapted from Singer (2006) preserves the original English source where it matched the validated instrument.*

*Instructions for participants. The questionnaire is anonymous. There are no right or wrong answers. Please respond to every item. The estimated completion time is 15–20 minutes. Mark only one response per item, unless otherwise indicated.*

## Part 1. Sociodemographic and workplace characteristics (10 items)

1.1. Sex: ☐ Female ☐ Male ☐ Prefer not to say

1.2. Age (years): ____

1.3. Total years of work experience in the healthcare system: ____

1.4. Years of work experience at the current workplace: ____

1.5. Highest completed level of education: ☐ Secondary medical school ☐ Bachelor (BSN) ☐ Master (MSN) ☐ Doctorate (PhD)

1.6. Hospital department of employment: ☐ Surgery ☐ Internal medicine ☐ Gynaecology/obstetrics ☐ Neurology ☐ Paediatrics ☐ Intensive care ☐ Anaesthesiology ☐ Psychiatry ☐ Other: ______

1.7. Shift pattern: ☐ Mornings only ☐ Two shifts ☐ Three shifts ☐ 12-hour shifts ☐ On-call

1.8. Nursing organisation model on your ward: ☐ Functional ☐ Team

1.9. Position: ☐ Registered nurse / nursing technician ☐ Senior nurse / unit lead ☐ Other: ______

1.10. Average weekly working hours: ☐ Up to 40 ☐ 40–59 ☐ 60 or more

## Part 2. Patient safety culture (39 items)

*Source for Sections 2.1–2.6: items adapted from the "Patient Safety Culture in the Hospital Survey" (Šklebar, 2014). Items marked with an asterisk (*) are reverse-coded.*

### 2.1. Interpersonal relationships (16 items)

*Please indicate the extent to which you agree or disagree with the following statements about your workplace/ward.*

| Statement | 1 — Strongly disagree | 2 — Disagree | 3 — Neither agree nor disagree | 4 — Agree | 5 — Strongly agree |
|---|---|---|---|---|---|
| 1. On our ward, we support each other. | ☐ | ☐ | ☐ | ☐ | ☐ |
| 2. We have enough staff to perform the required tasks. | ☐ | ☐ | ☐ | ☐ | ☐ |
| 3. When a lot of work has to be done in a short time, we work together as a team to get the job done. | ☐ | ☐ | ☐ | ☐ | ☐ |
| 4. On our ward, we respect each other. | ☐ | ☐ | ☐ | ☐ | ☐ |
| 5. On our ward, we work longer hours than is desirable from the standpoint of patient safety. | ☐ | ☐ | ☐ | ☐ | ☐ |
| 6. We actively take measures to improve patient safety. | ☐ | ☐ | ☐ | ☐ | ☐ |
| 7. We use more temporary staff than is necessary to provide the best possible care. | ☐ | ☐ | ☐ | ☐ | ☐ |
| 8. We feel that our errors are used against us.* (reverse-coded) | ☐ | ☐ | ☐ | ☐ | ☐ |
| 9. Errors on our ward lead to positive changes. | ☐ | ☐ | ☐ | ☐ | ☐ |
| 10. It is just by chance that more serious errors do not happen on our ward.* (reverse-coded) | ☐ | ☐ | ☐ | ☐ | ☐ |
| 11. When part of the ward becomes overloaded with work, we step in to help. | ☐ | ☐ | ☐ | ☐ | ☐ |
| 12. When an adverse event is reported, we feel it is directed at the person rather than at the problem.* (reverse-coded) | ☐ | ☐ | ☐ | ☐ | ☐ |
| 13. We never sacrifice patient safety in order to get more work done. | ☐ | ☐ | ☐ | ☐ | ☐ |
| 14. We are afraid that the errors we have made will remain in our personnel file.* (reverse-coded) | ☐ | ☐ | ☐ | ☐ | ☐ |
| 15. On this ward we have problems with patient safety.* (reverse-coded) | ☐ | ☐ | ☐ | ☐ | ☐ |
| 16. Our procedures and work organisation effectively prevent errors. | ☐ | ☐ | ☐ | ☐ | ☐ |

## 2.2. Your supervisor/manager (4 items)

*Please indicate the extent to which you agree or disagree with the following statements about your immediate supervisor/manager.*

| Statement | 1 — Strongly disagree | 2 — Disagree | 3 — Neither agree nor disagree | 4 — Agree | 5 — Strongly agree |
|---|---|---|---|---|---|
| 1. My supervisor/manager praises staff when work is performed in line with the established principles of patient safety. | ☐ | ☐ | ☐ | ☐ | ☐ |
| 2. My supervisor/manager takes staff suggestions for improving patient safety into account. | ☐ | ☐ | ☐ | ☐ | ☐ |
| 3. When workload increases, my supervisor/manager asks us to work faster, even at the expense of cutting corners.* (reverse-coded) | ☐ | ☐ | ☐ | ☐ | ☐ |
| 4. My supervisor/manager ignores recurrent patient safety problems.* (reverse-coded) | ☐ | ☐ | ☐ | ☐ | ☐ |

## 2.3. Communication (6 items)

*How frequently do the following situations occur in your workplace/ward?*

| Statement | 1 — Never | 2 — Rarely | 3 — Sometimes | 4 — Often | 5 — Always |
|---|---|---|---|---|---|
| 1. We receive feedback about changes implemented on the basis of adverse-event reports. | ☐ | ☐ | ☐ | ☐ | ☐ |
| 2. Staff freely speak up if they observe something that may have a negative effect on the patient's health. | ☐ | ☐ | ☐ | ☐ | ☐ |
| 3. We are informed about errors that occur on our ward. | ☐ | ☐ | ☐ | ☐ | ☐ |
| 4. Staff feel free to question the decisions or actions of those in authority. | ☐ | ☐ | ☐ | ☐ | ☐ |
| 5. On our ward we discuss ways to prevent the recurrence of errors. | ☐ | ☐ | ☐ | ☐ | ☐ |
| 6. Staff are afraid to ask questions when something does not seem right to them.* (reverse-coded) | ☐ | ☐ | ☐ | ☐ | ☐ |

## 2.4. Frequency of adverse-event reporting (3 items; main analysis used item 1)

*How often do you report adverse events when the following situations occur in your workplace/ward?*

| Statement | 1 — Never | 2 — Rarely | 3 — Sometimes | 4 — Often | 5 — Always |
|---|---|---|---|---|---|
| 1. When an error occurs but is detected and corrected before it can harm the patient, how often do you report it? | ☐ | ☐ | ☐ | ☐ | ☐ |
| 2. When an error occurs that has no potential for harm to the patient, how often do you report it? | ☐ | ☐ | ☐ | ☐ | ☐ |
| 3. When an error occurs that could harm the patient but did not, how often do you report it? | ☐ | ☐ | ☐ | ☐ | ☐ |

### 2.5. Management approach to patient safety (10 items)

*These statements refer to your hospital.*

| Statement | 1 — Strongly disagree | 2 — Disagree | 3 — Neither agree nor disagree | 4 — Agree | 5 — Strongly agree |
|---|---|---|---|---|---|
| 1. Hospital management creates a climate that promotes patient safety. | ☐ | ☐ | ☐ | ☐ | ☐ |
| 2. A lot of information is lost when patients are transferred from one ward to another.* (reverse-coded) | ☐ | ☐ | ☐ | ☐ | ☐ |
| 3. Hospital wards cooperate well with each other. | ☐ | ☐ | ☐ | ☐ | ☐ |
| 4. Important information given verbally about patient treatment is often lost during shift handovers.* (reverse-coded) | ☐ | ☐ | ☐ | ☐ | ☐ |
| 5. It is often unpleasant to work with staff from other hospital wards.* (reverse-coded) | ☐ | ☐ | ☐ | ☐ | ☐ |
| 6. Problems always arise during the exchange of information between hospital wards.* (reverse-coded) | ☐ | ☐ | ☐ | ☐ | ☐ |
| 7. The activities of hospital management show that patient safety is a top priority. | ☐ | ☐ | ☐ | ☐ | ☐ |
| 8. Hospital management appears to be interested in patient safety only after an adverse event has occurred.* (reverse-coded) | ☐ | ☐ | ☐ | ☐ | ☐ |
| 9. Hospital wards work together well to provide the best possible care to patients. | ☐ | ☐ | ☐ | ☐ | ☐ |
| 10. Shift changes are problematic for hospitalised patients.* (reverse-coded) | ☐ | ☐ | ☐ | ☐ | ☐ |

### 2.6. Self-perception of safety level and number of reports submitted

*Please rate patient safety at your workplace/ward (mark only one):*

☐ 1 — None ☐ 2 — Poor ☐ 3 — Acceptable ☐ 4 — Very good ☐ 5 — Excellent

*Indicate how many adverse-event reports you have completed and submitted in the past 12 months (mark only one):*

☐ None ☐ 1–2 ☐ 3–5 ☐ 6–10 ☐ 11–20 ☐ 21 or more ☐ Don't know / unknown

## Part 3. Personal experience with patient safety in the past 12 months (11 items)

*Source: Singer, S. J. (2006). Patient Safety Climate in Healthcare Organizations. Stanford University School of Medicine. Used with the author's written permission. Items marked with an asterisk (*) are reverse-coded.*

| Statement | 1 — Strongly disagree | 2 — Disagree | 3 — Neither agree nor disagree | 4 — Agree | 5 — Strongly agree | 6 — Not applicable |
|---|---|---|---|---|---|---|
| 1. I had adequate technical resources to provide safe patient care. | ☐ | ☐ | ☐ | ☐ | ☐ | ☐ |
| 2. I did not inform anyone about an error that had significant consequences and that no one else had noticed.* (reverse-coded) | ☐ | ☐ | ☐ | ☐ | ☐ | ☐ |
| 3. I learned how to do my job better by learning from my colleagues' errors. | ☐ | ☐ | ☐ | ☐ | ☐ | ☐ |
| 4. I had enough time to provide safe patient care. | ☐ | ☐ | ☐ | ☐ | ☐ | ☐ |
| 5. My formal education provided sufficient training to allow me to detect patient-safety problems. | ☐ | ☐ | ☐ | ☐ | ☐ | ☐ |

| Statement | 1 — Strongly disagree | 2 — Disagree | 3 — Neither agree nor disagree | 4 — Agree | 5 — Strongly agree | 6 — Not applicable |
|---|---|---|---|---|---|---|
| 6. I was rewarded for taking quick action to identify a serious error. | ☐ | ☐ | ☐ | ☐ | ☐ | ☐ |
| 7. My work was evaluated against defined safety standards. | ☐ | ☐ | ☐ | ☐ | ☐ | ☐ |
| 8. When I made an error, I was publicly shamed.* (reverse-coded) | ☐ | ☐ | ☐ | ☐ | ☐ | ☐ |
| 9. Staff felt free to speak up when they saw something that could negatively affect patient care. | ☐ | ☐ | ☐ | ☐ | ☐ | ☐ |
| 10. The procedure for tracking incidents and accidents on the ward was an effective way to identify causes. | ☐ | ☐ | ☐ | ☐ | ☐ | ☐ |
| 11. Intentional violations of standard operating procedures were rare on the ward. | ☐ | ☐ | ☐ | ☐ | ☐ | ☐ |

## Part 4. Frequency of nursing care practices that may negatively affect patient safety in the past 12 months (34 items)

*Source: Herak (2022); items developed on the basis of Delamont (2013), Friganović et al. (2020), Gurková et al. (2020), and Neuberg (2017). Please indicate, for each practice, how many times in the past 12 months you have observed it on your ward.*

### 4.1. Practices related to personal hygiene (8 items)

| Statement | 1 — None | 2 — 1–5 times | 3 — 6–10 times | 4 — 11 or more times |
|---|---|---|---|---|
| 4.1.1. Bathing patients routinely without observing the proper sequence of washing. | ☐ | ☐ | ☐ | ☐ |
| 4.1.2. Performing patient hygiene without using a privacy screen in a multi-bed room. | ☐ | ☐ | ☐ | ☐ |
| 4.1.3. Changing bed linen only when visibly soiled. | ☐ | ☐ | ☐ | ☐ |
| 4.1.4. Changing personal clothing only once a week. | ☐ | ☐ | ☐ | ☐ |
| 4.1.5. Neglecting skin and mucous-membrane care (not using creams or lotions). | ☐ | ☐ | ☐ | ☐ |
| 4.1.6. Leaving a patient in a soiled diaper for more than one hour. | ☐ | ☐ | ☐ | ☐ |
| 4.1.7. Leaving a patient on a bedpan/sani-chair for longer than necessary. | ☐ | ☐ | ☐ | ☐ |
| 4.1.8. Leaving a patient in one position for more than two hours. | ☐ | ☐ | ☐ | ☐ |

### 4.2. Practices related to medication administration (5 items)

| Statement | 1 — None | 2 — 1–5 times | 3 — 6–10 times | 4 — 11 or more times |
|---|---|---|---|---|
| 4.2.1. Administering a medication in the wrong dose. | ☐ | ☐ | ☐ | ☐ |
| 4.2.2. Administering a medication via the wrong route. | ☐ | ☐ | ☐ | ☐ |
| 4.2.3. Administering a medication at the wrong time. | ☐ | ☐ | ☐ | ☐ |
| 4.2.4. Administering a medication to the wrong patient. | ☐ | ☐ | ☐ | ☐ |
| 4.2.5. Failing to administer an ordered medication. | ☐ | ☐ | ☐ | ☐ |

### 4.3. Practices related to infection prevention (8 items)

| Statement | 1 — None | 2 — 1–5 times | 3 — 6–10 times | 4 — 11 or more times |
|---|---|---|---|---|
| 4.3.1. Irregular hand hygiene (washing and applying antiseptic) before and after a procedure. | ☐ | ☐ | ☐ | ☐ |
| 4.3.2. Not using gloves (latex, PVC, sterile) where the protocol requires them. | ☐ | ☐ | ☐ | ☐ |
| 4.3.3. Failing to use aseptic technique when drawing blood. | ☐ | ☐ | ☐ | ☐ |
| 4.3.4. Failing to use aseptic technique when inserting and caring for an intravenous cannula. | ☐ | ☐ | ☐ | ☐ |
| 4.3.5. Failing to use aseptic technique when administering intravenous therapy. | ☐ | ☐ | ☐ | ☐ |

| Statement | 1 — None | 2 — 1–5 times | 3 — 6–10 times | 4 — 11 or more times |
|---|---|---|---|---|
| 4.3.6. Failing to use aseptic technique when performing urinary catheterisation. | ☐ | ☐ | ☐ | ☐ |
| 4.3.7. Failing to use aseptic technique when caring for a central venous catheter. | ☐ | ☐ | ☐ | ☐ |
| 4.3.8. Irregular changing of the urinary catheter / leaving it in place longer than the protocol allows. | ☐ | ☐ | ☐ | ☐ |

## 4.4. Practices related to fall prevention (5 items)

| Statement | 1 — None | 2 — 1–5 times | 3 — 6–10 times | 4 — 11 or more times |
|---|---|---|---|---|
| 4.4.1. Not raising side rails on the bed. | ☐ | ☐ | ☐ | ☐ |
| 4.4.2. Excessive use of sedative medications. | ☐ | ☐ | ☐ | ☐ |
| 4.4.3. Not performing gradual mobilisation/verticalisation of the patient. | ☐ | ☐ | ☐ | ☐ |
| 4.4.4. Placing aids (glasses, walking stick, hearing aid) out of the patient's reach. | ☐ | ☐ | ☐ | ☐ |
| 4.4.5. Restraining a patient inappropriately. | ☐ | ☐ | ☐ | ☐ |

## 4.5. Practices related to food and fluid intake (3 items)

| Statement | 1 — None | 2 — 1–5 times | 3 — 6–10 times | 4 — 11 or more times |
|---|---|---|---|---|
| 4.5.1. Force-feeding (forcing the mouth open) when feeding by spoon. | ☐ | ☐ | ☐ | ☐ |
| 4.5.2. Inadequate feeding through a nasogastric tube/stoma (inappropriate rate, food consistency, failure to flush the stoma). | ☐ | ☐ | ☐ | ☐ |
| 4.5.3. Irregular care of the nasogastric tube/stoma site. | ☐ | ☐ | ☐ | ☐ |

## 4.6. Practices related to documentation and communication (5 items)

| Statement | 1 — None | 2 — 1–5 times | 3 — 6–10 times | 4 — 11 or more times |
|---|---|---|---|---|
| 4.6.1. Not performing patient identification (asking "What is your name?" or checking the wristband barcode) before nursing procedures (medication administration or specimen collection). | ☐ | ☐ | ☐ | ☐ |
| 4.6.2. Irregular documentation of nursing procedures in the nursing record. | ☐ | ☐ | ☐ | ☐ |
| 4.6.3. Not introducing oneself as a nurse/nursing technician on contact with the patient and when performing interventions. | ☐ | ☐ | ☐ | ☐ |
| 4.6.4. Not providing verbal instructions or descriptions of the interventions being performed on the patient. | ☐ | ☐ | ☐ | ☐ |
| 4.6.5. Not performing patient identification before medical procedures (surgical procedure, colonoscopy, ultrasound). | ☐ | ☐ | ☐ | ☐ |

## 4.7. Anonymity of adverse-event reporting (5 items)

*Please indicate the extent to which you agree or disagree with the following statements.*

| Statement | 1 — Strongly disagree | 2 — Disagree | 3 — Neither agree nor disagree | 4 — Agree | 5 — Strongly agree |
|---|---|---|---|---|---|
| 1. The anonymity of personal data positively affects the willingness to report an adverse event. | ☐ | ☐ | ☐ | ☐ | ☐ |
| 2. The omission of personal data in the report would lead to more frequent reporting of adverse events. | ☐ | ☐ | ☐ | ☐ | ☐ |
| 3. Anonymity of the report protects the reporter from possible negative consequences. | ☐ | ☐ | ☐ | ☐ | ☐ |
| 4. Anonymity reduces the perception that adverse-event reporting is directed against an individual. | ☐ | ☐ | ☐ | ☐ | ☐ |
| 5. The current adverse-event reporting system in my workplace ensures appropriate protection of personal data. | ☐ | ☐ | ☐ | ☐ | ☐ |

***Thank you for your participation.***

*Total number of items: 99 (10 sociodemographic + 39 patient safety culture + 11 personal safety experience + 34 nursing care practices + 5 anonymity statements). All items were closed-ended with a single response option. The instrument was administered on paper.*
